# Factors influencing non-communicable disease policy process in Sub-Saharan Africa: a scoping review

**DOI:** 10.1136/bmjph-2024-001409

**Published:** 2025-08-12

**Authors:** Enny Brouns, Chanelle Mulopo, Solange Mianda, Joy Mauti, Shannon McMahon, Connie Hoe, Bey-Marrie Schmidt

**Affiliations:** 1 Heidelberg Institute of Global Health, Ruprecht-Karls-Universität Heidelberg, Heidelberg, Germany; 2 School of Public Health, University of the Western Cape, Cape Town, South Africa; 3 Centre for Epidemic Response and Innovation, Stellenbosch University, Stellenbosch, Western Cape, South Africa; 4 School of Public Health, University of the Western Cape, Bellville, South Africa; 5 Institute of Public Health, Ruprecht Karls Universitat Heidelberg, Heidelberg, Germany; 6 International Health, Johns Hopkins University Bloomberg School of Public Health, Baltimore, Maryland, USA; 7 Johns Hopkins Bloomberg School of Public Health, Baltimore, Maryland, USA; 8 Health Systems Research Unit, South African Medical Research Council, Cape Town, South Africa

**Keywords:** Public Health, Systematic Review, Cardiovascular Diseases

## Abstract

**ABSTRACT:**

**Introduction:**

Non-communicable diseases (NCDs) have been the leading global cause of death for two decades, with a disproportionate impact on low- and middle-income countries. Despite the development of technical packages such as the WHO Best Buys, the adoption and implementation of NCD policies pose significant challenges. This scoping review explores the factors influencing the NCD policy process, including agenda setting, formulation, adoption, implementation and evaluation stages.

**Methods:**

This scoping review followed the methodological framework provided by Levac *et al*. To identify relevant studies for the scoping review, we searched the literature in the following databases: Web of Science and Scopus using PubMed. Reviewers independently screened titles, abstracts and full texts, and extracted data from the included studies. The results were collected using Excel and synthesised using descriptive numerical and thematic analysis.

**Results:**

The search yielded 7538 records, after screening for title, abstract and full text, 35 articles met the inclusion criteria for this review. Six different types of policy actors were identified, namely, (i) government, (ii) private sector, (iii) advocates, (iv) experts, (v) international partners, (vi) experts and (vii) general public. Policy actors used lobbying tactics to influence how the policy process was executed; however, it was unclear how the process of influence took place. We identified six barriers of the NCD policy process: (i) limited access to resources, (ii) limited reliable local data, (iii) role of the government, (iv) limited multisectoral collaboration, (v) limited infrastructure, (vi) knowledge and belief. Six facilitators of the NCD policy process were identified: (i) multisectoral approach, (ii) sufficient capacity and financial resources, (iii) access to reliable local evidence, (iv) strong advocacy, (v) existing infrastructures, and (vi) political will were reported.

**Conclusion:**

Findings from this review revealed a knowledge gap in understanding of the tactics used by actors to influence the policy process and the absence of evidence related to the evaluation of NCD policies in Sub-Saharan Africa.

**WHAT IS ALREADY KNOWN ON THIS TOPIC**

- WHO’s Regional Office in Africa foresees a shift in the leading cause of mortality by 2033, from a combination of communicable, maternal, neonatal and nutritional diseases to non-communicable diseases (NCDs).
- In Sub-Saharan Africa (SSA), the NCD policy process remains opaque, which limits scientific and pragmatic efforts to address this growing health challenge.

**WHAT THIS STUDY ADDS**

- This scoping review identified key factors influencing the NCD policy process in SSA. These key factors are categorised as policy actors, barriers and facilitators.
- Limited evidence: (i) on specific tactics used by actors to influence the policy process and (ii) evaluation of evidence-based NCD policies in the local context.

**HOW THIS STUDY MIGHT AFFECT RESEARCH, PRACTICE OR POLICY**

- Future research should explore how actors influence the policy process and assess the evaluation stage of the NCD policy process in SSA.

## Introduction

 A non-communicable disease (NCD) refers to a medical condition or illness that is inherently non-infectious and cannot be transmitted from one person to another.^1^ These diseases may manifest as chronic ailments with prolonged durations and gradual development, or they could lead to more abrupt fatalities, such as sudden strokes.^1^

As outlined by the WHO, the primary categories of NCDs encompass cardiovascular diseases (CVD) (eg, heart attacks and strokes), cancer, chronic respiratory diseases (CRD) (eg chronic obstructive pulmonary disease and asthma) and diabetes (DM).^1^ With an annual mortality percentage of more than 70%, NCDs have been the leading cause of death globally for the past 20 years.^2^

The rise of premature death caused by NCDs in low-income and middle-income countries (LMICs) is growing rapidly.^3^ In the last two decades, there has been a notable increase in the burden of NCDs in Sub-Saharan Africa (SSA). By 2030, NCDs are projected to surpass the combined impact of communicable, maternal, neonatal and nutritional diseases (CMNN) as the primary cause of mortality in the region.^4^

To address this growing pandemic, the WHO has issued various technical packages including the ‘Best Buys,’^5^ which outlines policy solutions that are economically efficient, rooted in empirical evidence and generate substantial returns on investment for governments to embrace.^5^ Although these cost-effective recommendations were published in 2017, their adoption and implementation have proven challenging.^6 7^

In this review, the term ‘policy process’ refers to a broader, more dynamic and realistic concept that captures the complex, non-linear interactions and negotiations influenced by political, social and institutional factors. It represents the overarching process in which policies evolve. In contrast, the ‘policy cycle’ offers a more structured, linear approach, dividing policy developments into five stages: agenda setting, policy formulation, decision-making or policy adoption, policy implementation and policy evaluation.^8^ The policy cycle simplifies this process for analytical purposes, but it is inherently part of the larger policy process.^9^ Together, these concepts provide complementary perspectives, balancing theoretical clarity with the complexity of real-world policy making.^9^

In SSA, the policy process, especially concerning NCDs, is characterised by opacity, with limited available knowledge. Existing reviews on the NCD policy process tend to have narrow scopes, concentrating on specific domains like a specific policy stage,^10 11^ risk factor,^12 13^ a specific country^14^ or an economic level.^15^ In contrast, this scoping review seeks to provide a comprehensive overview of existing evidence on this topic by synthesising information about the actors, facilitators and barriers influencing the entire NCD policy cycle in SSA.

## Methods

We conducted a scoping review informed by Levac *et al*’s framework.^16^ This framework builds on earlier methods for scoping reviews,^17^ emphasising the importance of matching the goals and questions of a review to its scope and techniques. We adopted the first five stages of the Levac *et al* framework; identifying the review question; identifying relevant studies; selecting relevant studies; charting the data; and collating, summarising and reporting the results; omitting the last and optional stage (stakeholder consultation) due to time constraints. This review aimed to explore the diverse factors (actors, barriers, facilitators) influencing NCD policy processes in SSA and to provide a nuanced understanding of how these factors interact with each other. The review was registered on Open Science Framework in February 2023 (https://doi.org/10.17605/OSF.IO/ZCQ5S).

### Identifying the review question

We used the population/concept/context (PCC) framework by the Joanna Briggs Institute^18^ to formulate the review question: What are the factors (actors, barriers, facilitators) influencing NCD policy cycle and how do these affect the policy process in SSA countries? The authors aimed to address the following sub-questions: (i) Which policy actors influence the various stages of the NCD policy process in SSA? (ii) What barriers and facilitators influence the NCD policy process in SSA? (iii) How do actors, facilitators and barriers influence the NCD policy process in SSA?

### Identifying the relevant studies

Two review authors (EB, CM) worked with a librarian at the University of the Western Cape to develop the search strategy (online supplemental annex 1). The search strategy was developed in PubMed through an iterative process, where keywords and medical subject headings (MeSH) terms were identified from known studies and preliminary searches. The remaining searches were carried out in Web of Science and Scopus using the PubMed search strategy but adjusted to the requirements of these databases. These three databases were selected because they would likely yield a large amount of relevant papers. A comprehensive search was conducted between 6 February and 17 February 2023.

### Selecting relevant studies

In the review process, six authors (EB, CM, SM, JM, SAM, BMS) independently screened titles and abstracts to determine eligibility for full-text screening in Covidence. CM compiled all search records into Covidence, a review production software, with the subsequent removal of duplicates.^19^ Covidence facilitated the automatic retrieval of open-access full-text articles; EB and CM manually obtained the remaining articles. Full-text articles were screened in duplicate by four review authors (EB, CM, SM, BMS). Two review authors (EB, JM) reviewed the selected full-text papers (EB reviewed CM, SM, BMS and JM reviewed EB). Conflicts during screening were resolved by two review authors (JM, CM).

The eligibility criteria used to select relevant studies were shaped by the PCC framework.^18^ Table 1 highlights the eligibility criteria. Moreover, the Preferred Reporting Items for Systematic Reviews and Meta-Analyses extension for Scoping Reviews (PRISMA-ScR) was used to guide the reporting of the review.^20^

**Table 1:** Eligibility criteria

Eligibility	Inclusion criteria	Exclusion criteria
Domain	Literature on NCDs: including diabetes mellitus, cancers, chronic respiratory disease, heart disease and NCD risk factors such as hypertension, obesity, tobacco, nutrition, physical inactivity, alcohol	N/A
Concept	Policy process (one or more stage(s))^8^ Agenda setting, to identify issues that require government attention. Formulation, to develop the policy’s structure. Adoption, to accept or deny the policy. Implementation, to put the policy into action. Evaluation, to examine the effects of the ongoing policy in an empirical, objective and systematic method.	N/A
Context	Sub-Saharan Africa	Studies published in other settings
Publication date	N/A	N/A
Publication language	Studies published in English	Studies published in other languages
Methods	Original studies such as interviews, focus group discussions, surveys, questionnaires and observational studies. Review studies; non-methodologically	Protocols, review studies; methodologically Grey literature (due to time and resource constraints)

N/A, not applicable; NCD, non-communicable diseases.

### Charting the data

A data extraction template was designed in Microsoft Excel for the extraction of relevant data from the included studies. The template included 19 categories as presented in table 2. Data extraction was conducted into two phases: In phase one, the first reviewers (EB, CM, SM and BMS) independently extracted and organised the relevant data into Excel sheets. In phase two, the second reviewers (EB, JM) reviewed the extracted data on relevance, completeness and accuracy. Conflicts were discussed and solved between the first and second reviewers. Once data extraction was completed, EB and CM were able to filter according to the individual categories extracted to compare and synthesise data from the included studies. No quality assessment was performed, as is consistent with the methodological framework of scoping reviews.^17^

**Table 2:** Data extraction sheet

Data extraction sheet	
1	Code
2	First reviewer
3	Second reviewer
4	Conflict reviewer
5	Title of the paper
6	Year of publication
7	Authors
8	Method
9	Data collection strategy
10	Study objectives
11	Setting
12	Stages of the policy cycle
13	NCD elements
14	Barriers
15	Facilitators
16	Policy actors
17	Key message
18	Implications
19	Additional information

NCD, non-communicable disease.

### Collating, summarising and reporting results

**Figure 1:**
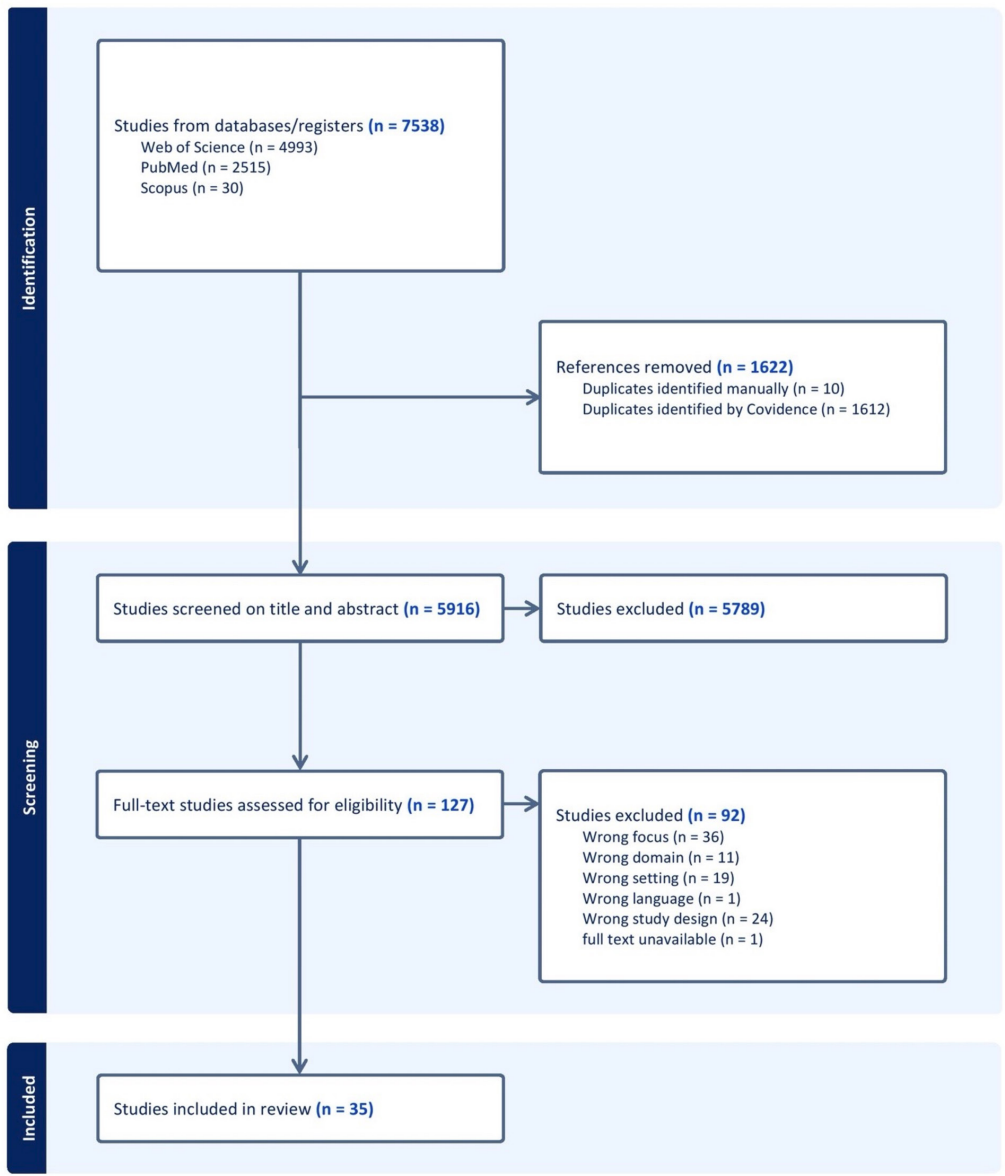
A PRISMA flowchart of the screening process of included studies. PRISMA, Preferred Reporting Items for Systematic Reviews and Meta-Analyses.

EB carried out a descriptive numerical and thematic analysis. The analysis process was continuously checked by the rest of the review team (CM, SM, JM, SAM, BMS). The numerical analysis entailed descriptively quantifying the characteristics of the included studies, while the thematic analysis followed the approach by Braun and Clarke^21^ to collate and synthesise the findings. First, familiarisation with the data took place by reading and re-reading the extractions of included studies as well as making notes to capture early impressions. Next, initial codes were generated by extracting smaller sections from the data, following the guidelines outlined in the data extraction sheet (figure 1). The data were manually coded by the first review author (EB), and two review authors (CM, JM) checked the coding process for accuracy and consistency. Additionally, a MIRO board^22^ (a digital collaborative whiteboard) was created to organise codes into themes (EB). Next, two review authors (CM, JM) refined the preliminary themes based on the review objectives. The final step entailed a list of the refined themes and subthemes, and their descriptions.

## Results

### Results of the search

Searching the electronic databases yielded 7538 records as illustrated in figure 1. Following the elimination of duplicates and independent screening of titles and abstracts, 127 studies qualified for full-text screening. During the full-text screening, 92 papers were excluded as they did not meet the inclusion criteria; they were not available in English (n=1), not focused on NCDs (n=11), not conducted in SSA (n=19), not original research (n=24) or they did not primarily address the policy cycle (n=36). The remaining 35 papers were included in the review.

### Description of the included studies

Of the 35 included studies, most were qualitative studies (n=30),^23^,^52^ the remaining were quantitative (n=3)^53^,^55^ and mixed methods (n=2)^56 57^ respectively. The Walt and Gilson policy triangle framework^58^ was used in 11 studies,^24 30 31 34 37 41 43 46 47 49^ followed by the John Kingdon’s multiple streams framework^59^ (n=3)^25 32 51^ and a thematic analysis framework (n=2).^36 40^ In nine studies,^26 27 33 39 42 45 54 55^ no framework was found. Apart from the two regional studies conducted across SSA,^42 45^ the NCD policy cycle, or a portion thereof, has been investigated in about half of the countries within the region. Seven studies were conducted in two or more (up to 15) countries^23 30 31 44 48 54 57^ and the majority (n=26) were focused on a single country.^24^,^2932^ 12 studies focused on a single stage of the policy cycle,^23^,^2630 33 41 47 53 55^ whereas, the majority (n=23) of studies focused on multiple stages^27^,^2931 32 34^ in various combinations. Overall, 24 papers researched the implementation stage,^2526 28 29 31 32 35 36 39 40 43 44 48^,^52 56 57^ and 19 studies described the formulation stage.^2427 28 30 31 35^,^37 40 41 44 46 47 49 52 54^ In 16 studies, agenda setting was mentioned,^2932 35 40 42 49^,^52 54^ 12 studies investigated the adoption stage^23 25 31 32 37 39 43 48 51^ and nine studies the evaluation stage.^33 35 36 42 53 55^ Only three studies looked at the entire policy process.^34 38 45^

Furthermore, this review highlighted eight key elements, reflecting the main NCDs such as CVD, CRD, cancers and DM, along with their associated risk factors contributing to NCD development (tobacco use, harmful use of alcohol, physical inactivity and diet-related risk factors). Among the four primary NCDs, only one study specifically addressed CVD^27^ and two studies focused on DM.^42 46^ However, CRD and cancers were not mentioned in any of the included studies. All risk factors including tobacco use (n=7),^27 32 35 44 45 53 55^ harmful use of alcohol (n=1),^24^ physical inactivity (n=2)^41 47^ and diet-related risk factors (n=12) were examined.^2325 27 33 37 39 41 43 48^,^50 54^ Moreover, two additional types of focus were found. The first, denoted as, ‘All risk-factors’ encapsulated studies where NCD risk factors were either undefined or included at least the primary NCD risk factors. Notably, only one study reported within this category.^34^ The second, captured as, ‘All NCDs’ referring to studies that did not define the NCDs clearly or that included at least the four main NCDs (CVD, CRD, DM, cancers) (n=12).^2628^,^31 36 37 40 50 52 56 57^ Additional information on the included studies can be found in online supplemental annex 2.

### Policy actors

Drawing on a thematic analysis, six categories of NCD policy actors in SSA were identified (table 3), with the majority predominantly influencing the implementation stage.^2325^,^29 31 32 34^ Except for the category ‘general public’, which primarily played a role in the agenda-setting stage.^32 40 42 50^

**Table 3:** Barriers, facilitators and policy actors per policy cycle stage

Barriers	Facilitators	Policy actors
Agenda setting		
Limited access to resources^2934 35 42 50^,^52^	Multisectoral approach^29 32 34 38 42 49 50^	Government^2932 34 35 38 40 42 45 49^,^52 54^
Limited reliable local evidence^2934 35 38 40 50^,^52^	Access to sufficient capacity and financial resources^29 42^	Private sector^32 34 40 45 51^
Role of the government^29 32 35 38 40 50 52 54^	Access to reliable local evidence^29 32 35 38 45 51 52^	Advocates^29 32 34 38 40 42 45 50 52^
Limited multisectoral approach^2935 38 40 42 45 50^,^52^	Advocacy^29 32 34 35 38 40 49^	International and intergovernmental organisations^32 38 40 42 49 50 52 54^
Insufficient infrastructure^29 35 38 40 42 50^	Existing infrastructures^29 35 38 49 50 52^	Experts^29 45 50 52^
Knowledge and belief^29 42 51 52^	Political will^29 35 38 40 42 45 54^	General public^32 40 42 50^
Formulation		
Limited access to resources^24 27 28 30 31 35 36 41 44 46 52^	Multisectoral approach^24 27 31 35 36 38 40 44 49^	Government^2728 30 31 34^,^38 40 41 44^
Limited reliable local evidence^28 34 35 38 40 46 52^	Sufficient capacity and financial resources^24 44 46^	Private sector^24 28 31 35 37 40 44 45^
Role of the government^28 30 31 35 36 38 40 41 46 52 54^	Access to reliable local evidence^27 35 37 38 44 45 52^	Advocates^2728 31 34 36^,^38 40 44^
Limited multisectoral approach^2428 30 31 35 40 41 44^,^47 52^	Advocacy^34 35 37 38 40 49^	International and intergovernmental organisations^27 34 36 38 40 44 49 52 54^
Insufficient infrastructure^28 35 38 40 46 47^	Existing Infrastructures^27 35 38 49 52^	Experts^31 36 44 45 52^
Knowledge and belief^24 27 31 36 41 52^	Political will^27 31 35 38 40 44 45 54^	General public^24 40^
Adoption		
Limited access to resources^31 34 51^	Multisectoral approach^23 31 32 38 43^	Government^2325 31 32 34 37^,^39 43 45 48 51^
Limited reliable local evidence^34 38 48 51^	Sufficient capacity and financial resources^23^	Private sector^25 31 32 37 39 43 45 48 51^
Role of the government^25 31 32 38 43 48^	Access to reliable local evidence^23 32 37 38 43 45 51^	Advocates^25 31 32 34 37 38 45^
Limited multisectoral approach^23 25 31 38 45 51^	Advocacy^23 32 34 37 38 43 48^	International and intergovernmental organisations^32 34 38 48^
Insufficient infrastructure^23 38 45 48^	Existing infrastructures^23 38 43 48^	Experts^31 45^
Knowledge and belief^31 43 48 51^	Political will^23 31 38 45 48^	General public^32^
Implementation		
Limited access to resources^26^,^2931 34^	Multisectoral approach^23 26 27 29 31 32 35 38 40 43 44 46 49 50^	Government^2325^,^29 31 32 34^
Limited reliable local evidence^2829 34 35 38 40 46 48 50^,^52^	Sufficient capacity and financial resources^23 29 44 46^	Private sector^2526 28 31 32 35 39 40 43^,^45 48 50^
Role of the government^25 26 28 29 31 32 35 36 38 40 43 46 48 50 52^	Access to reliable local evidence^2327 29 32 35 38 43^,^45 51 52^	Advocates^25^,^2931 32 34 36 38 40 44^
Limited multisectoral approach^2325 26 28 29 31 35 38 40 45 46 50^,^52^	Advocacy^23 29 32 34 35 38 40 43 48 49^	International and intergovernmental organisations^2627 32 34 36 38 40 44 48^,^50 52^
Insufficient infrastructure^23 26 28 29 35 38 40 46 48 50^	Existing infrastructures^2326 27 29 35 38 43 48^,^50 52^	Experts^29 31 36 44 45 50 52 56 57^
Knowledge and belief^26 27 29 31 36 43 48 51 52 56^	Political will^23 27 29 31 35 38 40 44 45 48^	General public^32 40 50^
Evaluation		
Limited access to resources^34^,^3642^	Multisectoral approach^35 36 38 42^	Government^33^,^3638 42 45 53^
Limited reliable local evidence^34 35 38^	Sufficient capacity and financial resources^42^	Private sector^35 45^
Role of the government^35 36 38^	Access to reliable local evidence^35 38 45^	Advocates^33 34 36 38 42 45^
Limited multisectoral approach^35 38 42 45^	Advocacy^34 35 38^	International and intergovernmental organisations^34 36 38 42^
Insufficient infrastructure^33 35 38 42^	Existing infrastructures^35 38 53^	Experts^33 36 45 55^
Knowledge and belief^36 42 55^	Political will^35 38 42 45^	General public^42^

#### Government

Most of the included studies emphasised the government’s pivotal role in shaping the policy process. Specifically, 24 studies highlighted the Ministry of Health (MoH) as a significant influencer,^2527^,^32 35^ particularly in the implementation stage,^2527^,^29 31 32 35 36 38 40 46 48^ with notable influence on agenda setting^2023 26 29 40^,^43 45^ and formulation.^1819 21 22 26^,^29 31 32 37 38 40 43 45^ Other government departments (such as the Ministries of Finance, Education, Urban Planning, Agriculture and Trade, Sports and Physical Education, Decentralisation, Youth Affairs, External Relations, Communication, Justice, and Transport) primarily influenced policy formulation.^30 35 41 46 47 49 54^

Information supporting Table 3 is available in the online supplemental material.

#### Private sector

In most studies, the private sector, defined as businesses not government-controlled, is identified as a significant stakeholder in the NCD policy process. The private sector influenced all stages, but 11 studies specifically discussed the influence on the implementation stage^25 26 28 31 32 35 39 43 44 48 51^ and seven on the adoption stage.^25 31 37 39 43 48 51^ Only four studies reported on the private sector’s influence on agenda setting and seven studies on policy formulation. Some studies^24 25 32 37 39 43 45^ mentioned specific private commercial actors, such as the sugar, alcohol, tobacco, pharmaceutical or soft drinks industries, while others (n=8) did not specify any particular private sector actor.^26 28 36 38 40 42 48 51^

#### Advocates

Advocacy groups (n=21)^25^,^2931 32 34 36 38 40 44^ supported a political or social cause including civil society, media/journalists, non-governmental organisations (NGOs) and patient groups. Civil society primarily influenced policy implementation (n=9),^2528 29 31 34 36 44^,^46^ whereas, media/journalism actions influenced agenda setting (n=6).^32 38 40 42 45 51^ Local NGOs were found to be influential across different stages of the policy cycle, with a predominant focus on policy implementation. Most studies did not name the NGOs involved in the policy process.^26 31 36 52 56^

#### International partners

International partners, defined as international and intergovernmental organisations, were discussed in 14 included studies. Notably, 10 of the included studies identified the WHO as a key actor in the NCD policy process, with a primary influence on agenda setting.^2729 32 35^,^38 40 50 52^ Other organisations mentioned include The World Bank, Southern African Development Community (SADC) and WHO Framework Alliance.^27 32 38 49 54^ Two studies specifically addressed the influence of international funders; one discussed the influence of international funders across all stages,^34^ and the other discussed the influence of international funders in four stages: agenda setting, formulation, implementation and evaluation.^36^

#### Experts

The review showed that limited evidence is available on the role of NCD experts, including academic institutions, educational officers, community health workers, clinicians, nutritionists and representatives of medical institutions. Most studies showed the experts’ influence across single or multiple policy stages,^2930 33 36 44 50 52 55^,^57^ while one study identified influence across all stages.^45^ Academia played a significant role in policy formulation and implementation,^31 36 44 45 52 55^ one study also focused on agenda setting.^52^ Five studies^29 46 50 52 56^ reported that health experts particularly played a role in agenda setting and policy implementation. Additionally, three studies addressed non-health experts, such as teachers and educational officers, exploring their roles in agenda setting, policy formulation or policy implementation.^33 55 56^

#### General public

The general public, identified in five studies, primarily influenced policy formulation, followed by agenda setting.^24 32 40 42 50^ Additionally, two studies considered traditional leaders as potential stakeholders, mainly in the agenda-setting stage.^42 50^

#### Policy actors’ influence

There was little information available in the included studies about the influence of policy actors. Only a few studies (n=14) discussed lobbying tactics used by policy actors to influence the policy process.^2325 30 31 34 36^,^39 43 46 48 51 54^ Lobbying tactics were used, for example, by industry actors, to protest the sugar-sweetened beverage (SSB) tax.^23 25 37 39 43 48^ On the other hand, lobbying was also used by advocates,^31 51^ such as civil society, for the formulation of various NCD prevention legislations and interventions.^34 46^ In the case of South Africa, advocates implemented a national campaign, lobbying against tobacco and for a free-of-charge Quit-line.^30^ Moreover, within government departments, tactics were employed to establish collaborations with other stakeholders (such as other government departments, advocates and international partners) in order to secure NCD funding^38^ or oppose initiatives, such as derailing tax increases.^31 49 51^

### Barriers

This review identified six barriers to the NCD policy process in SSA. The majority of studies focused on addressing barriers within the implementation stage of the policy process, indicating a greater emphasis on this stage compared with others.^2325 26 28^,^30 32 34 36 38^

#### Limited access to resources

A common barrier described in the included studies was limited access to resources (n=18).^2426^,^31 34^ Eight studies described the correlation between limited resources and overreliance on global aid, influencing the agenda setting, formulation and adoption stage of the policy cycle.^24, 31, 36, 38, 44, 46, 50, 52^ Additionally, 10 studies also reported that insufficient human resources^1217 20 23 29 37^,^40 45^ and limited access to equipment and/or supplies^30 36 37 39 45^ were barriers to some stages of the policy cycle. Insufficient human resources led to ineffectively addressing NCDs, constraints in coordination during policy formulation, delay in policy development and limited implementation ability.^1718 20 29 37^,^40^ Limited access to equipment and/or supplies negatively influenced the implementation stage due to insufficient access to technical capacity, drugs, health education and medical supplies.^30 36 39 45^

#### Limited reliable local evidence

Limited reliable local evidence (n=11)^2829 34 38 40 46 48 50^,^52^ that was accurate and timeous was a challenge for priority setting.^29 50 52^ Some studies stated that local evidence should be provided in the formulation stage to decrease the reliance on donor-led research and improve the policy content by grounding it to the country’s needs.^28 38 46^ Local evidence was also needed to adopt policies, for instance, evidence on the effectiveness of SSB tax could convince decision makers to support such a policy.^48 51^

#### The role of the government

Changes in governmental structures and/or workforce could have led to limited political will, commitment and leadership (n=12).^2426 28^,^30 35 36 38 43 46 48 50^ Competing priorities and conflicting interests within government (n=10)^28 30 31 35 40 41 43 50 52 54^ could have led to NCDs not being prioritised because of greater focus on other health issues.^40 41 50 52^ Conflict of interest was a key barrier in the first three stages of the policy cycle. This predominantly occurred between the domains of health and economic growth, which may have led to a shift in power dynamics, benefiting several private sectors.^25 31 35 43^ Despite the support of advocates and civil society, it remained challenging for decision-makers to push NCDs on the agenda, formulate NCD policies and gain approval on these policies.^25 28 31 35 43 54^ Then, political change (n=4)^29 32 38 46^ affected the length of the policy development and led to dysfunctional departments due to rivalling parties.^38 46^

#### Limited multi-sectoral collaboration

Another barrier to the NCD policy process was limited multisectoral collaboration due to (i) inadequate management of the multisectoral approach (MSA) process (n=9),^24^,^2628 29 38 44 51 52^ for instance, during agenda setting, this could have led to ad hoc decision-making.^50^ Consequently, prioritising immediate needs over crucial issues. Furthermore, delays in the implementation of NCD initiatives were caused by inefficient management of working groups during priority setting.^29 52^ Six studies delved into (ii) stakeholder perceptions,^31 40 41 45 47 50^ revealing policymakers being labelled as ‘out of touch with reality,’ particularly affecting the agenda-setting stage.^50^ This disconnect between policymakers and the general population led to the prioritisation of issues that exclusively impacted the more affluent segments of society.^50^ Other challenges reported in the six studies included limited shared understanding of goals, divergent stakeholder expectations and weak framing of NCD problems.^41 44 47^ Seven studies described the (iii) absence of key stakeholders^28 30 35 38 44 46 47^ in all stages of the policy cycle as challenging. In Togo and South Africa, key actors critically reflected on their absence in the formulation stage, when they were asked to participate during the implementation of the policy.^45^ Various studies described ‘one sector involvement’, where policy development was predominantly driven by the health sector.^35 46^ Other barriers relating to MSA were (iv) insufficient commitment and cooperation among stakeholders (n=6)^28 29 31 35 41 44^ and (v) industry interference which involved actions such as hindering data sharing, product promotion and power pressure on other stakeholders (n=7).^23 24 30 35 40 42 45^

#### Insufficient infrastructure

Eight of the included studies discussed limited systems, primarily highlighting challenges within the health system.^2326 29 42 46^,^48 50^ For example, that an underfunded health system, inadequate screening programmes, weak health information systems and poor monitoring frameworks affect the policy implementation stage.^26 29 42 46 47 50 51^ Furthermore, the absence of policies and/or guidelines was described in six studies,^26 28 33 35 39 40^ including tobacco control, addressing NCDs, domestic guidelines and enforcement regulations affecting the implementation and evaluation stage.^26 28 33 35 38 40^

#### Knowledge and belief

Insufficient knowledge and limited public awareness on NCDs among decision-makers and government representatives contributed to challenges in both the formulation and adoption stages.^2429 31 36 41 43 48 51 55^,^57^ Moreover, cultural barriers stemming from the societal approval of unhealthy habits or within work environments,^24 26 27 42 52^ influenced by power dynamics and beliefs,^25 31^ pose additional obstacles during policy development and adoption. These obstacles manifested in delays and disruptions to the process.

### Facilitators

This review has identified six facilitators of the NCD policy process in SSA; these were primarily discussed in the context of the implementation stage of the policy process.^2326 27 29 32 34 36 38 40 43^,^46 48 50 52^

#### Multi-sectoral approach

Stakeholder engagement played an important role in the MSA and came across as a significant facilitator of the NCD policy process, influencing global, political and public engagement.^2627 32 35 40 42^,^44 49 50^ The inclusion of various actors had positive effects on policy development, including DM management, alcohol policy and NCD strategic plans.^24 26 30 31 38 44 46^ Moreover, stakeholder engagement positively influenced agenda setting, policy formulation and implementation, through strong collaboration across government departments, expert groups and steering committees enhancing policy formulation.^35 36 38 44 48 49^ Collaboration between stakeholders and organisations in day-to-day tasks played a crucial role in the implementation stage, supported by framing the prevention of NCDs as a common benefit.^23 26 44 48 49^

#### Sufficient capacity and financial resources

Sufficient capacity and financial resources affected all the stages of the policy cycle.^23 24 29 42 44 46^ For instance, efficient financial resource utilisation and tax revenue contribute to building technical capacity and local expertise during the implementation stage.^23 29 44 46^ Additionally, available resources supported agenda setting and formulation stages, contributing to a well-equipped health system with technical capacity, local expertise and workshops.^24 29 42 44^

#### Access to reliable local evidence

The use of reliable local evidence was identified as a facilitator across the entire NCD policy process.^2327 29 32 35 37 38 43^,^45 51 52^ Local evidence particularly influenced agenda setting by challenging opposition to SSB tax, triggering political responses to the tobacco epidemic and justifying the need for a national baseline survey on NCD risk factors.^29 32 37 38 50^ The influence of local evidence extended to policy formulation and implementation, with examples from the Seychelles and positive effects on policy adoption of SSB tax.^2327 43^,^45 50^ One study highlighted the leading role of epidemiological data on tobacco use in formulating tobacco control policies in Cameroon.^35^

#### Advocacy

Lobbying emerged as the predominant advocacy tactic discussed, influencing public awareness and agenda-setting initiatives on reducing high-fat diets or tobacco use.^29 40 49^ Public awareness, fuelled by knowledge and concern, heightened pressure for policy adoption during the adoption stage.^32 43^ Media and public speakers played pivotal roles in advocating for NCD prevention policies, particularly shaping the agenda-setting stage by crafting compelling headlines and mobilising public support.^32 34 35 37 40 42 48^ Furthermore, international support helped to address health priorities during agenda setting,^35 38^ while public support played a crucial role in influencing policy adoption and implementation.^23 48^ Public support played a crucial role in overcoming resistance from opposing stakeholders and exerting pressure on decision-makers throughout the policy process.^23 48^

#### Existing infrastructures

Existing infrastructures merely focused on health, tax and enforcement, supporting policy implementation by comprehensive legal and regulatory systems, existing laws for tax collection and well-established health, administration, enforcement and/or surveillance systems.^2327 35 48^,^50^ The availability of international NCD policies and/or guidelines served as a framework and provided guidance for national strategic planning and execution.^29 35 38 48 52^ Four studies reported on the positive effect on the formulation stage; for example, global policies such as ‘WHO’s Best Buys’ and recommendations suggested by the WHO framework convention on tobacco control (FCTC) contributed to the successful development of NCD strategic plans and/or policies on tobacco control.^35 38 44 48^ These global policies also played an important role in the implementation stage.^29 44 48^

#### Political will

Strong political will mainly affected policy formulation,^18 26 29 31 35 36^ especially during the development of tobacco control policies.^27 35 38 40 44^ Moreover, two studies highlighted the efficacy of political will in addressing and prioritised the burden of NCDs during the agenda-setting stage.^29 42^ Political commitment enabled the implementation of NCDs in national health policies and tobacco control policies.^32^ Another example was found in the agenda-setting stage where political commitment was a facilitator to address tobacco control issues and identify the need for a health standard of the Ghanaian population.^32 35 49^ One study found that political will influenced the successful adoption of SSB tax at national and regional levels.^23^ Several studies identified political leadership as a facilitator that positively influenced policy development, for example, leadership is demonstrated through target setting or by challenging a policy that predominantly served the industry sector.^27 31 43^

## Discussion

This scoping review provided an overview of existing evidence on the actors, facilitators and barriers influencing the NCD policy process in SSA.

Government, private sector, advocates, international partners, experts and the general public were identified as the key actors in the NCD policy process. Similar policy actors were identified in other regions of the world.^1160^,^65^ This review showed that key actors used tactics such as lobbying and grassroots mobilisation to exert influence on the policy process.^2325 30 31 34 36^,^39 43 46 48 51 54^ Additionally, a related strategy known as coalition building was also identified as being used in this context.^30 51^ There was little evidence on the extent of influence NCD policy actors had on the policy process.

This review revealed that limited financial resources are a significant barrier across different stages of the NCD policy process.^2426^,^31 34^ However, a few of the included studies explored this dimension, suggesting that financial availability alone may not be sufficient as a facilitator. It often requires simultaneous alignment with other facilitators, such as political will or a multisectoral approach, to achieve optimal outcomes.

Numerous barriers were identified in this review. These barriers are similar to those identified in a study conducted in Nepal on implementing a national multisectoral action plan for preventing and controlling NCDs.^14^ Comparable barriers persist across other public health domains in the region.^66^ Both studies also noted a scarcity of contact persons within departments, attributing it to the high turnover of government workers.^14 66^

The facilitators identified in this study align with those identified in another study conducted in SSA on translating evidence into policy.^67^ In this review, MSA was identified particularly as a strong facilitator affecting the policy process through multistakeholder engagement, inclusion and collaboration on the NCD policy process. Similar findings were reported in one study conducted in Ethiopia on NCD policy and strategy gaps in the reduction of behavioural risk factors, and another study in Tanzania on public health concern alongside a global initiative on NCDs. Both studies underscored the significance of collaborative efforts, engagement with national and international partners, and the establishment of an efficient multisectoral and intersectoral coordination mechanism.^68 69^

Few studies focused on the evaluation stage of the policy cycle despite the fact that it is essential to informing decision-making on the continuation or adjustment of existing policies and for future policy considerations.^70^ This finding is consistent with other studies conducted in other regions of the world. Allen *et al*, for example, described the lack of published evaluations of well-established NCD interventions in LMICs.^71^

### Study limitations

This scoping review has several limitations that should be acknowledged. The reliance on three major databases excluded grey literature and non-English studies, potentially limiting insights from interdisciplinary fields like economics and development studies and under-representing non-English-speaking regions, such as francophone Africa. Additionally, the focus on the selected NCDs excluded other important categories like mental health conditions. Similarly, the emphasis on lifestyle risk factors overlooks structural (eg, poverty-related) drivers that are critical in rural African contexts. Despite these limitations, this review provides valuable insights and highlights key gaps to guide future research and policy development.

## Conclusion

This comprehensive review sheds light on the multifaceted dynamics surrounding the NCD policy process in SSA. By analysing the roles of various actors, identifying barriers and highlighting facilitators, it provides valuable insights into shaping targeted interventions and guiding future research efforts. Through this clarification, the review establishes a foundational framework for addressing prevailing gaps and enhancing the effectiveness of NCD policy formulation and implementation in the region.

Moving forward, it is imperative that subsequent research endeavours prioritise a thorough exploration of the influence exerted by individual policy actors throughout the NCD policy process. Understanding the strategic manoeuvres employed by these actors is crucial for gaining a nuanced understanding of the intricacies within the policy landscape. Furthermore, there is an urgent need to delve into the evaluation of NCD policies, an area that remains largely unexplored, to facilitate evidence-informed decision-making and improve public health outcomes. Furthermore, stakeholder consultations emerge as a vital component in corroborating and enriching the findings of this review. Incorporating insights from diverse stakeholders, including policymakers, healthcare professionals and affected communities, is essential for refining our understanding of the nuances inherent in the NCD policy terrain. By leveraging the insights provided in this review, stakeholders can collaborate to enhance the efficacy of the NCD policy process in SSA. Such collaborative efforts hold the potential to yield tangible advancements in public health outcomes and mitigate the burden of NCDs across the region.

In conclusion, this review not only highlights existing challenges but also lays the groundwork for transformative interventions aimed at positively impacting the health and well-being of populations in SSA.

## Supplementary material

10.1136/bmjph-2024-001409 online supplemental table 1

10.1136/bmjph-2024-001409 online supplemental file 1

## Data Availability

All data relevant to the study are included in the article or uploaded as supplementary information.
