# Supplementary material for: Factors influencing non-communicable disease policy process in Sub-Saharan Africa: a scoping review
Source: BMJ Public Health. 2025 Aug 12;3(2):e001409. doi: 10.1136/bmjph-2024-001409 (PMC12352196; doi:10.1136/bmjph-2024-001409)
Supplement: online supplemental file 1 [file bmjph-3-2-s002.docx]

## **Annex 1: Search String**

**Search string PubMed**

| No. | Keywords | Search string | Hits |
| --- | --- | --- | --- |
| #1 | Non communicable disease | "noncommunicable diseases"[MeSH Terms] OR noncommunicable diseases[Text Word] OR noncommunicable disease*[Text Word] OR NCD[Text Word] OR NCDs[Text Word] OR NCD’s[Text Word] | 11,567 |
| #2 | Policy, Policy making, Regulations | "policy"[MeSH Terms] OR policy[Text Word] OR policies[Text Word] OR "Policy Making"[Mesh] OR regulation*[Text Word] OR policy transfer[Text Word] OR policy implementation[Text Word] OR policy adoption[Text Word] OR policy formulation[Text Word] OR policy adoption[Text Word] OR agenda setting[Text Word] | 2,066,039 |
| #3 | NCD Policy | "noncommunicable disease policy[Text Word] OR NCDs policy[Text Word] OR NCDs policy process[Text Word] | 964 |
| #4 | NCD Policy, Policy | #3 OR #4 | 2,066,039 |
| #5 | NCDs, Policy | #1 AND #4 | 2515 |

**Search string Web of Science**

| No. | Keywords | Search string | Hits |
| --- | --- | --- | --- |
| #1 | Non communicable disease | (ALL = (noncommunicable disease OR noncommunicable diseases OR NCD OR NCDs OR NCD’s) | 28,047 |
| #2 | Policy, Policy making, Regulations | Policy OR policy OR policies OR Policy Making OR regulation OR policy transfer OR policy implementation OR policy adoption OR policy formulation OR policy adoption OR agenda setting | FAIL |
| #3 | Policy, Policy making, Regulations | (ALL = (Policy OR Policies)) | 1,608,218 |
| #4 | NCD Policy | (ALL= noncommunicable disease policy OR NCDs policy OR NCDs policy process) | 3,579 |
| #5 | NCD Policy | #3 AND #4 | 3,004 |
| #6 | NCDs, Policy | #1 AND #5 | 4993 |

**Search string Scopus**

| No. | Keywords | Search string | Hits |
| --- | --- | --- | --- |
| #1 | Non communicable disease | noncommunicable AND disease OR noncommunicable AND diseases OR NCD OR NCDs OR NCD’s | 54,076 |
| #2 | Policy, Policy making, Regulations | policy OR policy OR policies OR policy AND making OR regulation OR policy AND transfer OR policy AND implementation OR policy AND adoption OR policy AND formulation OR policy AND adoption OR agenda AND setting | 192,931 |
| #3 | NCD Policy | noncommunicable AND disease AND policy OR ncds AND policy OR ncds AND policy AND process | 6,198 |
| #4 | NCD Policy, Policy | #3 OR #4 | 2,066,039 |
| #5 | NCDs, Policy | #1 AND #4 | 30 |

**Annex 2: Description of the dataset**

| **Author, year** | **Title** | **Method** | **Approach** | **Framework** | **Countries** | **Policy cycle** | **Element** |
| --- | --- | --- | --- | --- | --- | --- | --- |
| (Abdool Karim et al., 2021) | The legal feasibility of adopting a sugar-sweetened beverage tax in seven sub-Saharan African countries | Qual | Document review | Other | Botswana, Kenya, Namibia, Rwanda, Tanzania, Uganda & Zambia | Adoption | Diet-related risk factors |
| (Abiona, Oluwasanu  and Oladepo, 2019) | Analysis of alcohol policy in Nigeria: multi-sectoral action and the integration of the WHO "best-buy" interventions. | Qual | Document review & IDI | Walt and Gilson framework of policy analysis | Nigeria | Formulation | Alcohol |
| (Ahaibwe et al., 2021) | Barriers to, and facilitators of, the adoption of a sugar sweetened beverage tax to prevent non-communicable diseases in Uganda: a policy landscape analysis. | Qual | Policy analysis & IDI | John Kingdon’s multi streams Framework | Uganda | Adoption Implementation | Diet-related risk factors |
| (Bosu, 2012) | A comprehensive review of the policy and programmatic response to chronic non-communicable disease in Ghana. | Qual | Document review | No framework found | Ghana | Implementation | All NCDs |
| (Bovet et al., 2010) | Addressing non-communicable diseases in the Seychelles: towards a comprehensive plan of action. | Qual | Commentary | No framework found | Seychelles | Formulation Implementation | Cardiovascular diseases  Diet-related risk factors  Tobacco use |
| (Bünder, Karekezi and Wirtz, 2021) | Governing industry involvement in the non-communicable disease response in Kenya. | Qual | Document review & IDI | Other | Kenya | Formulation Implementation | All NCDs |
| (Chelwa, van Walbeek  and Blecher, 2017) | Evaluating South Africa's tobacco control policy using a synthetic control method | Quant | the synthetic control method | Other | South Africa | Evaluation | Tobacco use |
| (Essue and Kapiriri, 2018) | The unfunded priorities: an evaluation of priority setting for noncommunicable disease control in Uganda. | Qual | Document review & IDI | Other | Uganda | Agenda Setting  Implementation | All NCDs |
| (Juma, Mapa-tassou, et al., 2018) | Multi-sectoral action in non-communicable disease prevention policy development in five African countries. | Qual | Document review & IDI | Walt and Gilson framework of policy analysis | South Africa, Cameroon, Kenya, Malawi & Nigeria | Formulation | All NCDs |
| (Juma, Mohamed, et al., 2018) | Non-communicable disease prevention policy process in five African countries. | Qual | IDI | Walt and Gilson framework of policy analysis | South Africa, Kenya, Cameroon, Nigeria & Malawi | Formulation Adoption Implementation | All NCDs |
| (Kusi-Ampofo, 2021) | Negotiating Change: Ideas, Institutions, and Political Actors in Tobacco Control Policy Making in Mauritius. | Qual | Document review & IDI | John Kingdon’s multi streams Framework | Mauritius | Agenda Setting Adoption Implementation | Tobacco use |
| (Laar et al., 2020) | Implementation of healthy food environment policies to prevent nutrition-related non-communicable diseases in Ghana: National experts' assessment of government action | Qual | Support indicators & Panel | No framework found | Ghana | Evaluation | Diet-related risk factors |
| (Lupafya et al., 2016) | Implementation of Policies and Strategies for Control of Noncommunicable Diseases in Malawi: Challenges and Opportunities. | Mixed | Semi structured Questionnaire | No framework found | Malawi | Implementation | All NCDs |
| (Mamka Anyona et al., 2014) | An Analysis of the Policy Environment Surrounding Noncommunicable Diseases Risk Factor Surveillance in Kenya. | Qual | Document review & IDI | Walt and Gilson framework of policy analysis | Kenya | All stages | All risk factors |
| (Mapa-Tassou et al., 2018) | Two decades of tobacco use prevention and control policies in Cameroon: results from the analysis of non-communicable disease prevention policies in Africa. | Qual | Document review, IDI & field observations | Walt and Gilson framework of policy analysis | Cameroon | Agenda Setting Formulation Implementation Evaluation | Tobacco use |
| (Meghani et al., 2021) | Curbing the Rise of Noncommunicable Diseases in Uganda: Perspectives of Policy Actors. | Qual | IDI | Thematic data analysis | Uganda | Formulation Implementation Evaluation | All NCDs |
| (Mukanu et al., 2017) | Nutrition related non-communicable diseases and sugar sweetened beverage policies: a landscape analysis in Zambia. | Qual | Document review & IDI | Walt and Gilson framework of policy analysis John Kingdon’s Framework | Zambia | All stages | All NCDs |
| (Mukanu et al., 2021) | Responding to non-communicable diseases in Zambia: a policy analysis. | Qual | Document review & IDI | Walt and Gilson framework of policy analysis | Zambia | Formulation Adoption | Diet-related risk factors |
| (Myers et al., 2017) | The history of the South African sugar industry illuminates deeply rooted obstacles for sugar reduction anti-obesity interventions | Qual | Document review | No framework found | South Afrcia | Adoption Implementation | Diet-related risk factors |
| (Ndinda et al., 2018) | The evolution of non-communicable diseases policies in post-apartheid South Africa. | Qual | Document review & IDI | Thematic data analysis | South africa | Agenda Setting  Formulation Implementation | All NCDs |
| (Oluwasanu, Oladunni and Oladepo, 2020) | Multisectoral approach and WHO 'Bestbuys' in Nigeria's nutrition and physical activity policies | Qual | Document review & IDI | Walt and Gilson framework of policy analysis | Nigeria | Formulation | Diet-related risk factors  Physical inactivity |
| (Renzaho, 2015) | The post-2015 development agenda for diabetes in sub-Saharan Africa: challenges and future directions. | Qual | Document review | No framework found | SSA | Agenda Setting  Evaluation | Diabetes Mellitus |
| (Ruhara et al., 2021) | Strengthening prevention of nutrition-related non-communicable diseases through sugar-sweetened beverages tax in Rwanda: a policy landscape analysis. | Qual | Document review & IDI | Walt and Gilson framework of policy analysis | Rwanda | Adoption Implementation | Diet-related risk factors |
| (Sanni et al., 2018) | Assessment of the multi-sectoral approach to tobacco control policies in South Africa and Togo. | Qual | Policy analysis & IDI | Other | South Africa & Togo | Formulation Implementation | Tobacco use |
| (Sanni et al., 2019) | Multi-Sectoral Approach to Noncommunicable Disease Prevention Policy in Sub-Saharan Africa: A Conceptual Framework for Analysis. | Qual | Policy analysis & IDI | No framework found | SSA | All stages | Tobacco use |
| (Shiroya et al., 2019) | Challenges in policy reforms for non-communicable diseases: the case of diabetes in Kenya. | Qual | Policy analysis & IDI | Walt and Gilson framework of policy analysis | Kenya | Formulation Implementation | Diabetes mellitus |
| (Shiroya et al., 2021) | Reorienting Primary Health Care Services for Non-Communicable Diseases: A Comparative Preparedness Assessment of Two Healthcare Networks in Malawi and Zambia. | Mixed | Survey & IDI | Other | Zambia & Malawi | Implementation | All NCDs |
| (Tatah et al., 2021) | Analysis of Cameroon's Sectoral Policies on Physical Activity for Noncommunicable Disease Prevention. | Qual | Policy analysis | Walt and Gilson framework of policy analysis | Cameroon | Formulation | Physical inactivity |
| (Thow et al., 2014) | Development, implementation and outcome of standards to restrict fatty meat in the food supply and prevent NCDs: learning from an innovative trade/food policy in Ghana. | Qual | Policy analysis & IDI | Walt and Gilson framework of policy analysis | Ghana | Agenda Setting Formulation Implementation | Diet-related risk factors |
| (Thow et al., 2015) | Regional trade and the nutrition transition: opportunities to strengthen NCD prevention policy in the Southern African Development Community. | Quant | Time series graphs of import data for soft drinks and snack foods | No framework found | South Afrcia, Mozambique, DRC, Tanzania, Zambia, Malawi, Angola, Zimbabe, Lesotho, Madagaskar, Swaziland, Botswana, Namibia, Seychelles & Mauritius | Agenda Setting  Formulation | Diet-related risk factors |
| (Thow et al., 2021) | The political economy of sugar-sweetened beverage taxation: an analysis from seven countries in sub-Saharan Africa. | Qual | Document review & IDI | Other | Botswana, Kenya, Namibia, Rwanda, Tanzania, Uganda & Zambia | Adoption Implementation | Diet-related risk factors |
| (Viswanathan et al., 2011) | Impact of a smoking ban in public places: a rapid assessment in the Seychelles | Quant | Surveys & questionnaires | No framework found | Seychelles | Evaluation | Tobacco use |
| (Wanjau et al., 2021) | Stakeholder perceptions of current practices and challenges in priority setting for non-communicable disease control in Kenya: a qualitative study. | Qual | FGD | Other | Kenya | Agenda Setting  Implementation | All NCDs |
| (Wanjohi et al., 2021) | Nutrition-related non-communicable disease and sugar-sweetened beverage policies: a landscape analysis in Kenya. | Qual | Document review & IDI | John Kingdon’s multi streams Framework | Kenya | Agenda Setting Adoption Implementation | Diet-related risk factors |
| (Witter et al., 2020) | Opportunities and challenges for delivering non-communicable disease management and services in fragile and post-conflict settings: perceptions of policy-makers and health providers in Sierra Leone. | Qual | Document review & IDI | Other | Sierra Leone | Agenda Setting  Formulation  Implementation | All NCDs |
